# The B Cell Adaptor Molecule Bam32 Is Critically Important for Optimal Antibody Response and Resistance to *Trypanosoma congolense* Infection in Mice

**DOI:** 10.1371/journal.pntd.0003716

**Published:** 2015-04-13

**Authors:** Chukwunonso Onyilagha, Ping Jia, Nipun Jayachandran, Sen Hou, Ifeoma Okwor, Shiby Kuriakose, Aaron Marshall, Jude E. Uzonna

**Affiliations:** 1 Department of Immunology, Faculty of Medicine, University of Manitoba, Winnipeg, Manitoba, Canada; 2 Department of Medical Microbiology, Faculty of Medicine, University of Manitoba, Winnipeg, Manitoba, Canada; New York University School of Medicine, UNITED STATES

## Abstract

**Background:**

Bam32, a 32 kDa adaptor molecule, plays important role in B cell receptor signalling, T cell receptor signalling and antibody affinity maturation in germinal centres. Since antibodies against trypanosome variant surface glycoproteins (VSG) are critically important for control of parasitemia, we hypothesized that Bam32 deficient (Bam32-/-) mice would be susceptible to *T*. *congolense infection*.

**Methodology/Principal Findings:**

We found that *T*. *congolense*-infected Bam32^-/-^ mice successfully control the first wave of parasitemia but then fail to control subsequent waves and ultimately succumb to their infection unlike wild type (WT) C57BL6 mice which are relatively resistant. Although infected Bam32^-/-^ mice had significantly higher hepatomegaly and splenomegaly, their serum AST and ALT levels were not different, suggesting that increased liver pathology may not be responsible for the increased susceptibility of Bam32^-/-^ mice to *T*. *congolense*. Using direct *ex vivo* flow cytometry and ELISA, we show that CD4+ T cells from infected Bam32^-/-^ mice produced significantly increased amounts of disease-exacerbating proinflammatory cytokines (including IFN-γ, TNF-α and IL-6). However, the percentages of regulatory T cells and IL-10-producing CD4+ cells were similar in infected WT and Bam32^-/-^ mice. While serum levels of parasite-specific IgM antibodies were normal, the levels of parasite-specific IgG, (particularly IgG1 and IgG2a) were significantly lower in Bam32^-/-^ mice throughout infection. This was associated with impaired germinal centre response in Bam32^-/-^ mice despite increased numbers of T follicular helper (Tfh) cells. Adoptive transfer studies indicate that intrinsic B cell defect was responsible for the enhanced susceptibility of Bam32^-/-^ mice to *T*. *congolense* infection.

**Conclusions/Significance:**

Collectively, our data show that Bam32 is important for optimal anti-trypanosome IgG antibody response and suppression of disease-promoting proinflammatory cytokines and its deficiency leads to inability to control *T*. *congolense* infection in mice.

## Author Summary

African trypanosomiasis continues to be a major threat to human health and economic development in sub-Saharan Africa. Despite intense studies, the immunopathogenesis of the disease remains poorly understood. Understanding the factors that regulate disease pathogenesis would be important in designing effective immunotherapeutic strategies. Here, we demonstrate that the B cell adaptor molecule, Bam32, contributes to optimum resistance to experimental *T*. *congolense* infection in mice because its deficiency negatively impacts optimal B cell responses including germinal centre formation and parasite-specific IgG responses *in vivo*. In addition, Bam32 deficiency significantly enhances proinflammatory cytokine production by splenic cells, suggesting that Bam32 may act as a negative regulator of cytokine gene expression following *T*. *congolense* infection. Collectively, these findings identify Bam32 as an indispensable molecule for optimal germinal centre formation, anti-trypanosome IgG antibody response and suppression of disease-promoting proinflammatory cytokines and its deficiency leads to inability to control *T*. *congolense* infection in mice.

## Introduction

African trypanosomiasis, also called sleeping sickness in man, is a deadly disease of humans and livestock caused by blood parasites belonging to the genus *Trypanosoma*. In human, the disease is caused by *Trypanosoma brucei gambiense* and *Trypanosoma brucei rhodesiense*; whereas the animal form of the disease is primarily caused by *Trypanosoma congolense*, *Trypanosoma vivax and Trypanosoma brucei brucei* with *T*. *congolense* being the most important [1]. According to the World Health Organization (WHO) report, an estimated 60 million people are at risk of getting the infection with 300,000 cases of the disease occurring annually [2]. However, this is a gross under estimation because only about 10% of the cases are appropriately diagnosed and treated [2]. The animal form of the disease poses a huge agricultural and economic problem in the affected region due to reduced animal yield [3]. It is estimated that elimination of the disease would spare Africa an estimated $4.5 billion yearly as a result of improved animal production [4].

The control of parasitemia and resistance to African trypanosomes in mice have been linked to early interferon gamma (IFN-γ) production, which is important for activating macrophages to produce nitric oxide that has both trypanostatic and trypanotoxic effects [5–9]. In addition, IFN-γ is also important for production of optimal amounts and isotypes of parasite-specific IgG antibodies that are important for resistance via enhanced phagocytosis and complement-mediated lysis [10–12]. However, uncontrolled production of IFN-γ and other proinflammatory cytokines (including tumor necrosis factor-α [TNF-α], IL-6, IL-1β and IL-12) has been incriminated as the major cause of death in the highly susceptible mice [13–17]. On the other hand, IL-10 plays a regulatory role in dampening the excessive proinflammatory cytokines produced during infection [13].

Bam32 is a 32 kDa B lymphocyte adaptor protein that plays an important role in B cell receptor (BCR) cross-linking-mediated downstream events [18] and has been shown to be expressed in B cells, T cells, dendritic cells and macrophages [19,20]. Bam32 has also been shown to be important in BCR internalization [21], BCR-induced signalling, B cell survival [22] and antigen presentation [23]. Upon B cell antigen receptor cross-linking, Bam32 is tyrosine-phosphorylated and has been shown to be associated with phospholipase Cγ2 in human B cell lines [18]. However, deficiency of Bam32 does not affect the development of B and T cells, but significantly impairs B cell proliferation following BCR cross linking [24]. Importantly, Bam32^-/-^ mice also show reduced T-independent type two (TI-II) B cell responses [25] and high susceptibility to *Streptococcus pneumoniae* infection due to defective antibody response [24]. Bam32 is also implicated in T cell receptor signalling and regulation of CD4^+^ T cell cytokine production [26,27].

A strong antibody response (especially IgG and its subclasses) resulting from germinal centre reaction is required for the clearance of many pathogens including African trypanosomes [28]. The germinal centre is an extensive area of B cell proliferation, somatic hypermutation, selection, and class switch recombination leading to production of various antibody isotypes with high antigen binding affinity [29]. Upon encountering their cognate antigen, B cells become activated and require help from other immune cells (especially CD4^+^ follicular T helper cells and dendritic cells) to initiate germinal centre formation [30]. The follicular CD4^+^ T helper cells (Tfh) provide signals to antigen-specific B cells that guide their survival, expansion or differentiation into high affinity antibody-producing cells. Thus, Tfh are indispensable for germinal centre response [29] and hence for optimal antibody-mediated immunity.

Since antibodies and T cell cytokine production are both required for *T*. *congolense* clearance in infected animals, we investigated the role of Bam32 in experimental African trypanosomiasis using Bam32^-/-^ mice. Our report shows than Bam32 deficiency leads to inability to control late waves of undulating parasitemia, increased production of disease exacerbating proinflammatory cytokines by immune cells, impaired germinal centre B cell response and significantly lower serum levels of trypanosome specific IgG antibodies leading to early death in the otherwise relatively resistant strain of mice.

## Materials and Methods

### Ethics statement

All experiments involving mice were approved by the University of Manitoba Animal Care Committee in accordance with the regulation of the Canadian Council on Animal Care (Protocol Number 12–072).

### Mice

Six to eight week-old female C57BL/6 mice and CD1 (outbreed Swiss white mouse) were purchased from the Central Animal Care Services (CACS), University of Manitoba, Winnipeg, Canada. The origin and phenotype of Bam32^-/-^ mice have been previously described [18]. B cell deficient (μMT) mice on the C57BL/6 background were obtained from The Jackson Laboratory (Bar Harbor, ME, USA). Bam32^-/-^ mice on C57BL/6 background (> 12 generations) were bred by the CACS and supplied as needed. The origin and phenotype of p110δ knock-in mice (p110δ KI) has been previously described [31]. The housing, handling and feeding of experimental animals were in accordance to the recommendations of the Canadian Council of Animal Care.

### Parasite and infection of mice

Throughout the experiment, *Trypanosoma congolense* (Trans Mara strain), variant antigenic type TC13 was used and the origin of this strain has been previously described [32]. CD1 mice were immunosuppressed intraperitoneally (i.p.) with cyclophosphamide (Cytoxan; 200 mg/kg) and after two days, infected i.p. with TC13 stabilates [32]. Three days after infection, mice were deeply anaesthetized by isoflourane and blood was collected by cardiac puncture and parasites were purified by anion-exchange chromatography by passing through DEAE-cellulose chamber [33]. Eluted parasites were washed in Tris-saline glucose (TSG), counted, resuspended in TSG containing 10% heat-inactivated FBS and diluted to the desired concentration (10^4^/ml). In all the experiments, mice were infected intraperitoneally (100 μl) with 10^3^ parasites.

### Estimation of parasitemia

To estimate parasitemia, a drop of blood was taken from the tail vein of each *T*. *congolense-*infected mouse unto a microscopic slide, covered with cover slip and parasitemia was determined by counting the number of parasites presents in at least 10 fields at 400x magnification of light microscope. During periods of heavy parasite load, estimation was done as described previously [34].

### Cell culture, direct ex vivo staining and flow cytometry

Following sacrifice, the spleens were made into single-cell suspensions and contaminating red blood cells were lysed with ACK lysis buffer. Cells were washed 2 times with PBS, resuspended at final concentration of 4 x 10^6^/ml in complete tissue culture medium (DMEM supplemented with 10% heat-inactivated fetal bovine serum, 2 mmol L-glutamine, 100 U/mL Penicillin and 100 μg/ml streptomycin), and plated at 1 mL/well in 24-well tissue culture plates (Falcon; VWR Edmonton, AB, Canada) in the presence or absence of whole trypanosome lysate (10^6^/ml parasite equivalent). After 3 days, the cell culture supernatant fluids were collected and stored at -20°C until analyzed for cytokines by ELISA. Some cells were directly stained *ex-vivo* for CD4 and CD25 expression and intracellularly for Foxp3 using the Tregs staining kit (eBioscience, San Diego, CA) in accordance with the manufacturer’s recommendations. Germinal centre B cell response was assessed by staining splenic cells with fluorochrome-conjugated antibodies against B220, GL7 and Fas (all purchased from eBiosciensce). Antibodies against CD4, PD1 and ICOS (eBiosciensce) were used to assess T follicular helper cells response while antibodies against CD19, Fas, GL7, CD80 and PD-L2 were used to assess memory B cells. In some experiments, cells were stimulated with phorbol myristic acetate (PMA; 50 ng/mL), ionomycin (500 ng/mL), and brefeldin A (BFA; 10 μg/mL) for 4 hrs and stained for CD4 surface expression and for intracellular cytokine (TNF-α, IFN-γ and IL-10) expression. After staining, all samples were washed routinely in FACS buffer, acquired using *BD FACS Canto II* cytometer (BD Bioscience, San Diego CA), and analysed using FlowJo software (BD Bioscence).

### Elisa and liver function test

The levels of IL-6, TNF-α, IL-10, and IFN-γ in the culture supernatant fluids were determined by sandwich ELISAs using antibody pairs purchased from BD Biosciences according to the manufacturer's suggested protocols. The sensitivities of the ELISAs were 15, 31, 15 and 7.5 pg/ml for IL-6, TNF-α, IL-10, and IFN-γ, respectively. Serum levels of alanine aminotransferase (ALT) and aspartate aminotransferase (AST) were measured using Stanbio kits (Stanbio Laboratory, Boerner, TX) according to the manufacturer’s suggested protocols.

### Immunofluorescence microscopy

The spleens from infected mice were collected on indicated days and embedded in OCT (Tissue-Tek, Torrence, CA) before being snap frozen in liquid nitrogen. The frozen sections were cut to 8–10 μm size and fixed with paraformaldehyde for 15 min, washed with PBS and air-dried. Sections were blocked for 30 min with mouse Fc Block, washed with PBS containing Tween 20 (0.05%) and stained for 1 hr at room temperature with antibody cocktail containing FITC-labelled anti-PNA (Vector laboratories, Burlington, ON), PE-labelled anti-CD4 and APC-labelled IgD (BD Biosciences). The slides were mounted in Prolong Gold anti-fade reagent (Molecular Probes) after washing with PBS and viewed with Zeiss AxioObserver Spinning Disk Confocal Microscope.

### Isolation and adoptive transfer of B cells into μMT mice

Mature B (CD19^+^) cells were isolated from the spleens of naïve WT or Bam32^-/-^ mice by negative selection using a mouse B cell enrichment kit (StemCell Technology, Vancouver, BC) according to the manufacturer’s suggested protocol. The purity of the enriched B cells was greater than 95% as assessed by flow cytometry. Enriched B cells were washed in complete medium, resuspended in PBS and approximately 30 million cells were injected into each mouse through the tail vein. Recipient mice were infected with *T*. *congolense* (10^3^) 48 hr after cell transfer.

### Statistics

Data are represented as means and Standard Error of Mean (SEM). Two-tailed Student'st-test or ANOVA were used to compare means and SEM between two groups using GraphPad Prism software. Differences were considered significant at *p* < 0.05.

## Results

### Deficiency of Bam32 leads to enhanced susceptibility to experimental *T*. *congolense* infection

**Fig 1:**
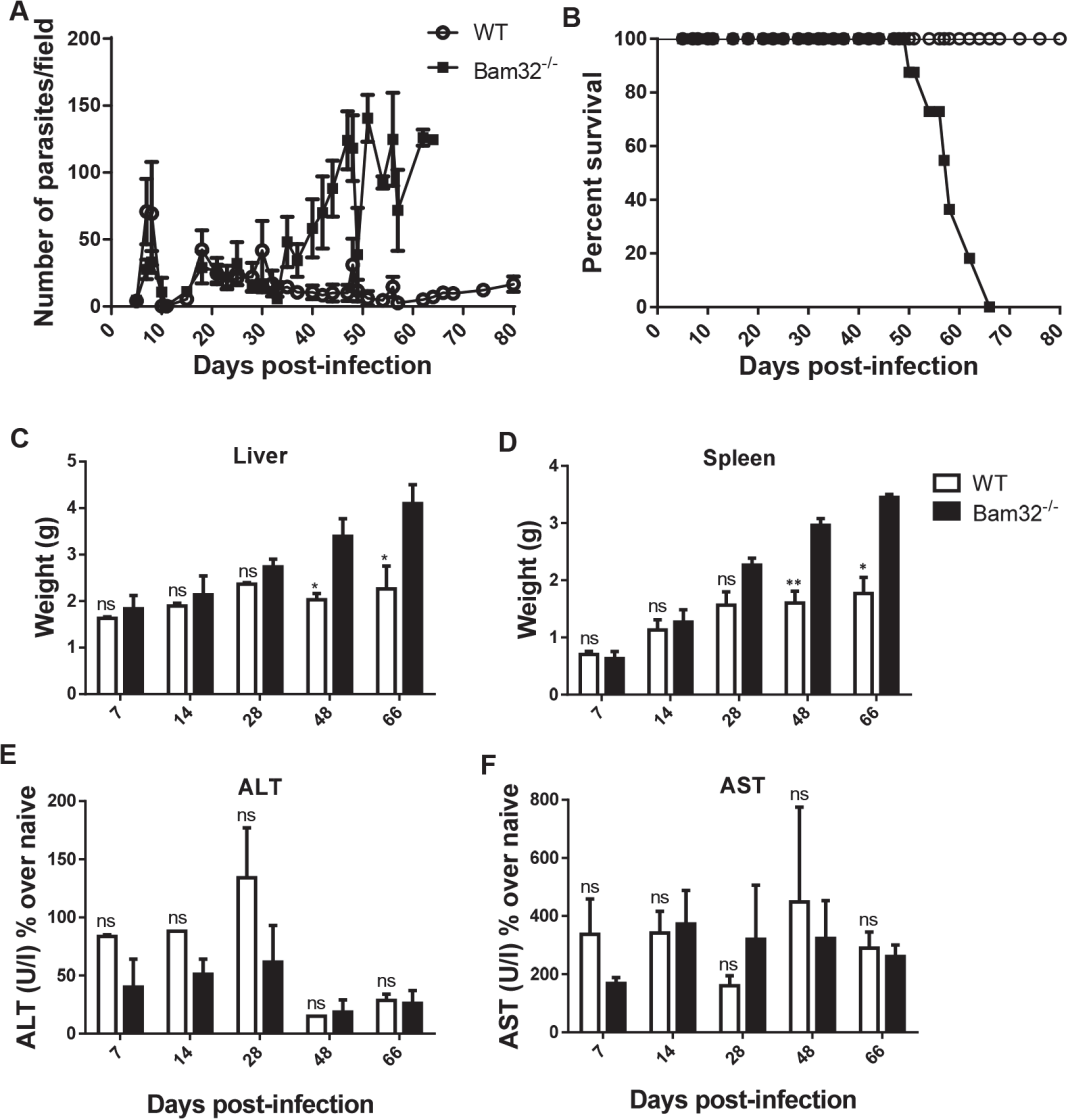
Bam32^-/-^ mice are susceptible to experimental *Trypanosoma congolense* infection. Groups of Bam32^-/-^ and WT C57BL/6 mice were infected intraperitoneally with 10^3^ *T*. *congolense*, clone TC13. Daily parasitemia (A) was estimated by direct counting of tail blood smears in at least 10 fields at 400X magnification. Infected mice were followed over time and the percentage survival (B) was recorded. At indicated times, infected mice were sacrificed and the liver (C) and spleen (D) weights were determined. In addition, serum levels of ALT (E) and AST (F) were determined. Results are representative of 3 (A and B) and 2 (C-F) different experiments (n = 3–5 mice per experiment) with similar outcome. ns, not significant. *, p < 0.05; **, p < 0.01

C57BL/6 mice are considered relatively resistant to *T*. *congolense* infection because they control several waves of undulating parasitemia and survive for more than 100 days post-infection before eventually dying [35]. Because Bam32^-/-^ mice exhibit increased susceptibility to infection with *Streptococcus pneumoniae* due to failure to generate opsonising IgG antibodies [24], we hypothesized that they will be susceptible to *T*. *congolense* infection. Therefore, we infected WT and Bam32^-/-^ mice intraperitoneally with 10^3^ *T*. *congolense* (TC13) and monitored parasitemia as well as survival period after infection. There were no differences in the prepatent period and level of parasitemia at the early phase of the infection between WT and Bam32^-/-^ mice (Fig 1A). However, Bam32^-/-^ mice developed fulminating parasitemia towards the late phase of the infection and succumbed to the infection (mean survival time 58 ± 9 days, Fig 1B). In contrast, all the WT mice survived with low parasitemia until day 80 when the experiment was terminated. These findings suggest that Bam32 molecule is important for effective control of parasitemia and survival in *T*. *congolense*-infected mice.

### Comparable serum levels of liver transaminases in infected WT and Bam32^-/-^ mice

*Trypanosoma congolense* infection in mice is associated with hepato-splenomegaly and an accompanying increase serum levels of liver enzymes including alanine aminotransferase (ALT) and aspartate aminotransferase (AST) [36]. This marked hepato-splenomegaly has been variously linked with increased pathology, activation and expansion of the reticuloendothelial system [37,38] and a compensatory extramedullary haematopoiesis [39,40]. Therefore, we determined whether there was a correlation between increased susceptibility of Bam32^-/-^ mice and liver pathology. Prior to day 28 post-infection, the liver and spleen sizes of WT and Bam32^-/-^ mice were indistinguishable (Fig 1C and 1D). However, from day 48 post-infection, the liver and spleens of infected Bam32^-/-^ mice were significantly larger than those of their WT counterpart mice. The increase in liver and spleen sizes coincided with the onset of increased and uncontrolled parasitemia in the late phase of the infection in Bam32^-/-^ mice (see Fig 1A). Interestingly, despite the significantly larger spleen and liver sizes in infected Bam32^-/-^ mice, there was no difference in serum levels of ALT (Fig 1E) and AST (Fig 1F) between infected WT and Bam32^-/-^ mice, suggesting that the hepatomegaly may not be associated with significant liver pathology and may not be directly responsible for the early death of infected Bam32^-/-^ mice.

### Enhanced production of disease exacerbating proinflammatory cytokines in infected Bam32^-/-^ mice

**Fig 2:**
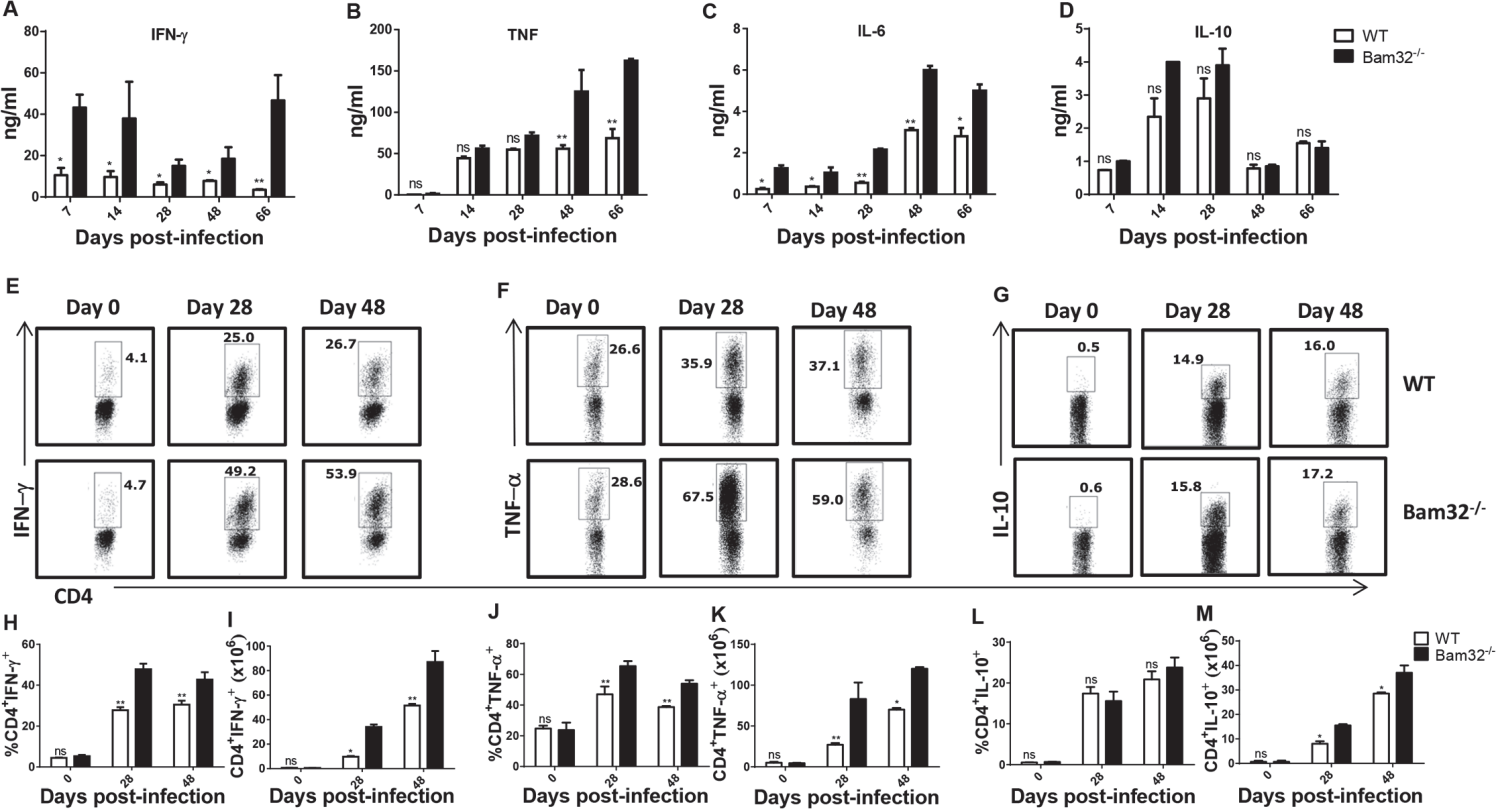
Increased production of proinflammatory cytokines by *T*. *congolense* infected Bam32^-/-^ mice. WT and Bam32^-/-^ mice were infected with 10^3^ *T*. *congolense*. At indicated times, infected mice were sacrificed, the spleen cells were cultured for 72 hr and the culture supernatant fluids were assayed for IFN-γ (A), TNF-α (B), IL-6 (C) and IL-10 (D) by ELISA. Some spleen cells were also directly stimulated *ex-vivo* with PMA, BFA and ionomycin for 3–5 hr, stained for intracellular expression of IFN-γ (E, H and I), TNF-α (F, J and K) and IL-10 (G, L and M), and analyzed by flow cytometry. Fig 2H–2M are bar graphs representing the means +/- standard error of the percentages (H, J and L) and absolute numbers (I, K and M) of CD4^+^ T cells that express IFN-γ (H and I), TNF-α (J and K) and IL-10 (L and M), respectively. Results are representative of 2 different experiments (n = 3–5 mice per experiment) with similar outcome. ns, not significant; *, p < 0.05; **, p < 0.01.

Previous studies have shown that susceptibility to *T*. *congolense* infection in mice is associated with the production of high levels of proinflammatory cytokines (including TNF-α, IL-6, IL-12, and IFN-γ) by spleen cells from infected mice leading to increased serum levels of the cytokines, systemic inflammatory response syndrome (SIRS) and death [14–17]. Because infected Bam32^-/-^ mice had higher and uncontrolled late phase parasitemia and succumbed to the infection significantly earlier than their infected WT counterpart mice (Fig 1A), we hypothesized that the levels of these cytokines would be significantly higher than those of WT mice. Throughout the infection, the levels of IFN-γ (Fig 2A), TNF-α (B) and IL-6 (C) in culture supernatant fluids of splenocytes from infected Bam32^-/-^ mice were significantly (p < 0.05–0.01) higher than those from WT mice, with the difference being more pronounced towards the time infected Bam32^-/-^ mice were unable to control parasitemia. Interestingly, there was no significant change in the secretion of IL-10 (Fig 2D), an anti-inflammatory cytokine that plays a critical role in dampening systemic inflammatory response syndrome, leading to survival in *T*. *congolense*-infected mice [13]. This pattern of cytokine response was also observed by intracellular cytokine staining where we found that the percentages and absolute numbers of IFN-γ (Fig 2E, 2H and 2I) and TNF-α (Fig 2F, 2J and 2K)-producing CD4^+^ cells from spleens of Bam32^-/-^ infected mice were significantly higher than those from infected WT mice. Interestingly and consistent with the ELISA data, the percentages of IL-10-producing CD4^+^ cells were not different between WT and Bam32^-/-^ mice, although the absolute numbers were higher than those from WT mice (Fig 2G, 2L and 2M), which could be due to higher splenomegaly during the late stage of infection (see Fig 1D). In addition and as shown in S1 Fig, CD3^+^ T cells were the major producers of IFN-γ, TNF-α and IL-10 in the spleens throughout the infection. Together, these results show that the absence of Bam32 molecule in mice infected with *T*. *congolense* leads to enhanced production of disease-exacerbating proinflammatory cytokines, which could account for the early death observed in infected Bam32^-/-^ mice.

### Regulatory T cells in infected Bam32^-/-^ mice

**Fig 3:**
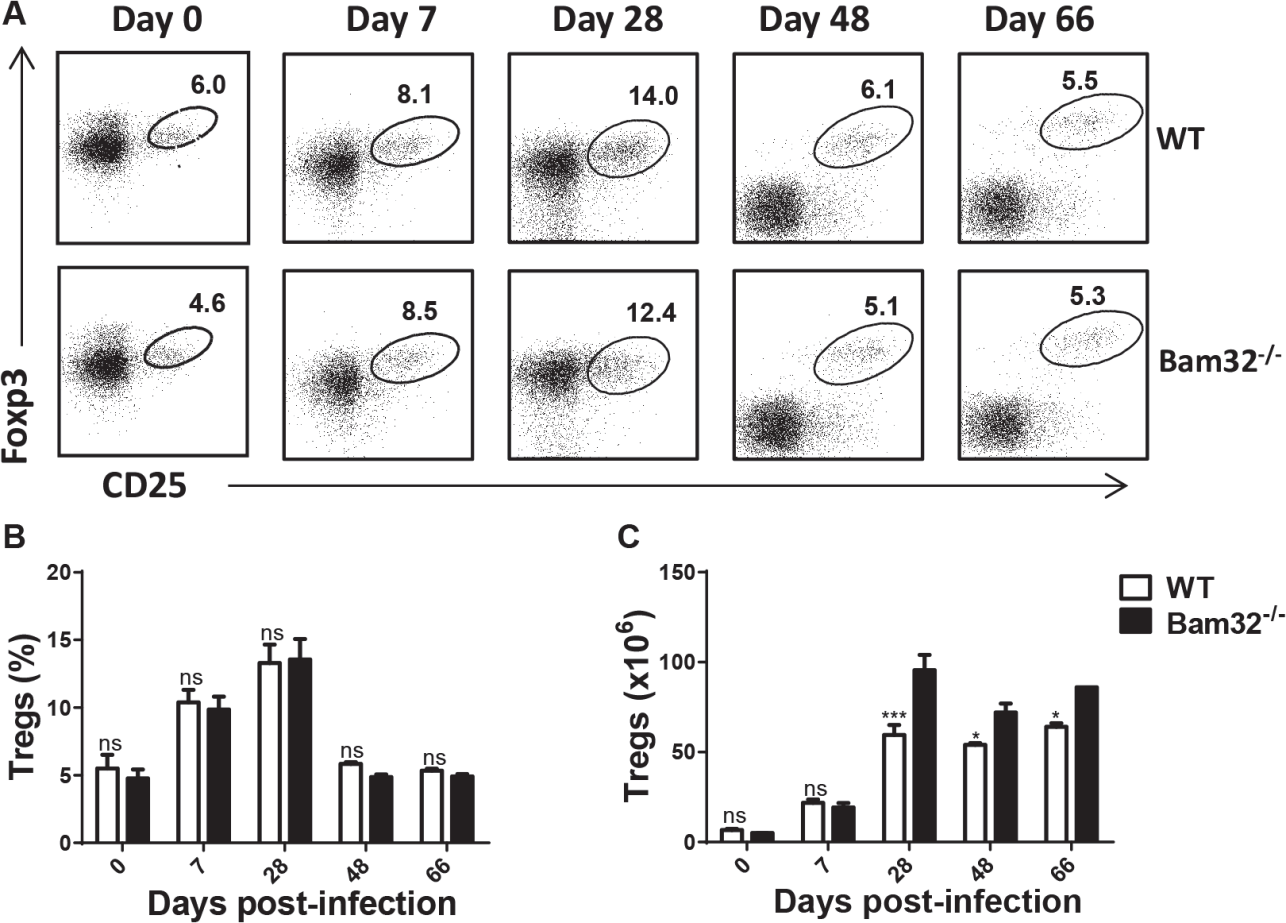
Comparable numbers of regulatory T cells (Tregs) in *T*. *congolense*-infected Bam32^-/-^ mice. Infected WT and Bam32^-/-^ mice were sacrificed at indicated times and their spleen cells were directly stained *ex vivo* for surface expression of CD4 and CD25 and intracellularly for Foxp3 expression. Dot plot (A) and bar chart (B) show the percentages of CD4^+^CD25^+^Foxp3^+^ cells at different times after infection. Cells were first gated for CD4 expression and then assessed for CD25 and Foxp3 expression. (C) Absolute numbers of CD4^+^CD25^+^Foxp3^+^ cells in the spleens of infected mice at different times after infection. Results are representative of 2 different experiments (n = 4–5 mice per experiment) with similar outcome. ns, not significant. *, p < 0.05; ***, p<0.001.

Naturally occurring regulatory T cells (Tregs) have been shown to play a pathogenic (disease-promoting) role in experimental African trypanosomiasis [41–43]. Because we found that Bam32^-/-^ mice were more susceptible than their WT counterpart mice, we investigated whether their spleens contained higher numbers of CD4^+^CD25^+^Foxp3^+^ T cells (Tregs). As shown in Fig 3, there was no difference in the pattern of expansion (Fig 3A) and percentages (Fig 3B) of Tregs in the spleen of WT and Bam32^-/-^ mice on the indicated days. However, the absolute numbers of Tregs (Fig 3C) were significantly higher in Bam32^-/-^ mice towards the later time points during the infection than those from WT mice, which may be a consequence of increased splenomegaly in these mice.

### Serum levels of trypanosome-specific antibodies in infected Bam32^-/-^ mice

**Fig 4:**
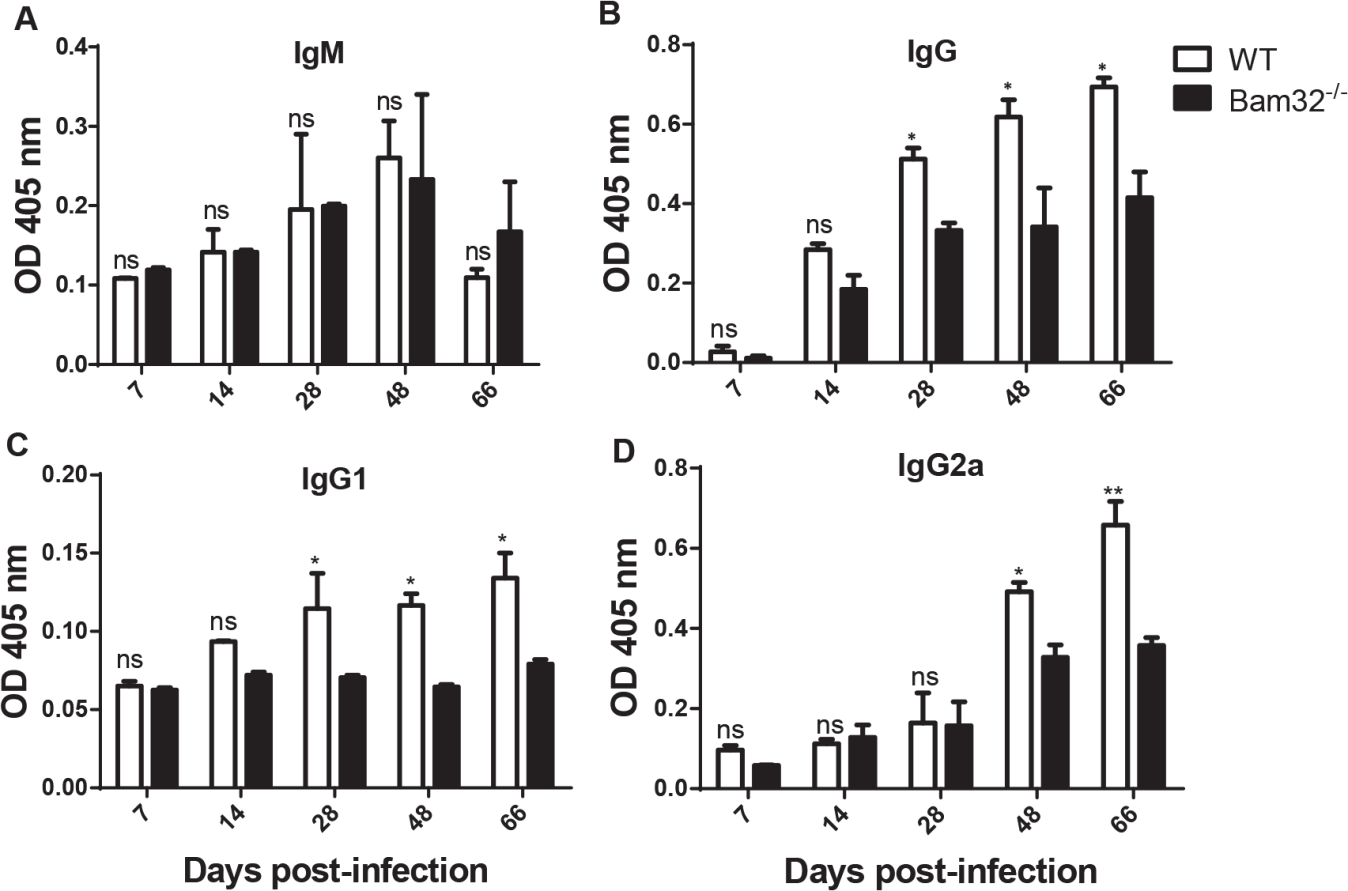
Impaired trypanosome-specific IgG response in *T*. *congolense* infected Bam32^-/-^ mice. WT and Bam32^-/-^ mice were infected with 10^3^ *T*. *congolense* and at indicated times, mice were sacrificed and their sera were analysed (change over naïve) for trypanosome-specific IgM (A), IgG (B), IgG1 (C) and IgG2a (D) antibodies using ELISA. Results are representative of 2 experiments (n = 3–5 mice per experiment) with similar outcome. ns, not significant; *, p < 0.05; **, p < 0.01

A strong IgG antibody response is important for survival of *T*. *congolense* infection in mice [28]. Bam32 is required for optimal affinity maturation in germinal centres leading to production of high affinity IgG1 and IgG2a antibodies [44] and for production of IgG3 antibodies in response to TI-II antigens [24]. Therefore, we investigated whether susceptibility of Bam32^-/-^ to *T*. *congolense* was related to defective IgG antibody response against the parasites. WT and Bam32^-/-^ mice infected with *T*. *congolense* were assessed for their serum levels of parasite-specific IgM and IgG antibodies at different time points. There was no difference in the kinetics and magnitude of IgM antibody responses in infected WT and Bam32^-/-^ mice (Fig 4A). In contrast, infected Bam32^-/-^ mice showed significantly reduced serum levels of trypanosome-specific IgG (Fig 4B), IgG1 (Fig 4C) and IgG2a (Fig 4D) starting from day 28 (IgG and IgG1) or 48 (IgG2a) post-infection, which corresponds to the onset of uncontrolled parasitemia in these mice. Collectively, these results indicate that deficiency of Bam32 leads to impaired production of trypanosome-specific IgG, IgG1 and IgG2a responses.

### Impaired germinal centre B cell response in the spleen of infected Bam32^-/-^ mice

**Fig 5:**
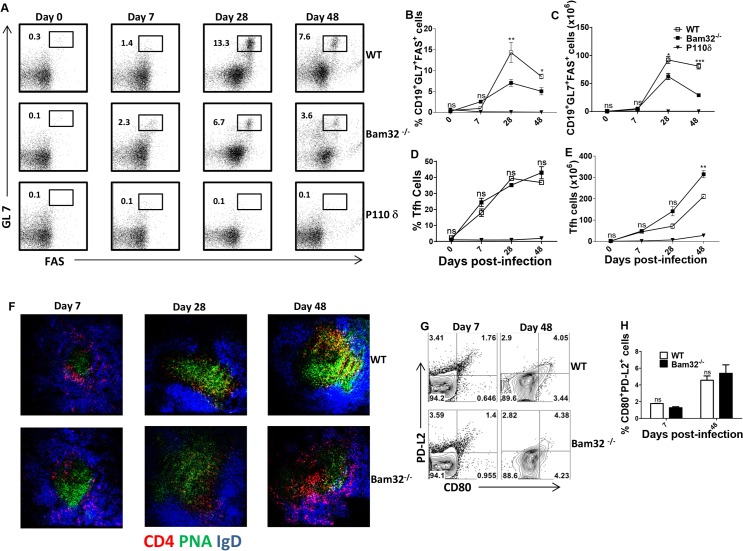
Impaired germinal centre B cell response in *T*. *congolense*-infected Bam32^-/-^ mice. WT and Bam32^-/-^ mice were infected with *T*. *congolense* and at the indicated days, the percentages (A, B and D) and absolute numbers (C and E) of germinal centre B cells (B220^+^GL7^+^Fas^+^, A-C) and T follicular helper cells (CD4^+^PD1^+^ICOS^+^, D and E) in the spleens of infected mice were assessed by flow cytometry. P110δ mutant mice that lack germinal centre response were added as extra control. Immunofluorescence staining of spleen sections from infected mice at different times post-infection is shown in Fig 5F. Representative contour plot (G) and bar graph (H) showing the percentages of CD19^+^GL7^-^Fas^-^CD80^+^PD-L2^+^ cells (memory B cells) and shown at different times after infection. Spleen cells from infected WT and Bam32^-/-^ mice were stained directly *ex vivo* for surface expression of CD19, GL7, Fas, CD80 and PD-L2 molecules. Thereafter percentage of CD80^+^PD-L2^+^ cells was then assessed by flow cytometry after gating on CD19^+^GL7^-^Fas^-^ population. Data shown are representative of 3 (A-F) and 1 (G and H) separate experiments (n = 4–5 mice per experiment) with similar outcome. *, p <0.05; **, p < 0.01; ***, p < 0.001; ns, not significant.

The reduced production of parasite-specific IgG1 and IgG2a antibodies in infected Bam32^-/-^ mice suggested a defect in germinal centre responses. Flow cytometric analysis show that the onset and magnitude of germinal centre B cell response (day 7 post-infection) were similar in infected WT and Bam32^-/-^ mice (Fig 5A). However, from day 28 post-infection, infected Bam32^-/-^ mice have significantly (p < 0.05–0.001) lower percentages (Fig 5A and 5B) and absolute numbers (Fig 5C) of germinal centre B cells compared to their WT counterpart mice. P110δ knock-in mice that lack the ability to form germinal centres [45,46] were included in the experiment to serve as additional control. We further performed immunofluorescence staining in order to validate the flow cytometry results. The data presented as Fig 5F showed that the onset of germinal centre formation in Bam32^-/-^ mice was comparable to that of WT mice. However, as infection progressed, the germinal centre structure in infected Bam32^-/-^ mice began to disintegrate, such that by day 48 post-infection, very few follicular B cells were evident (Fig 5F). Interestingly, there were no differences in the percentages of T follicular helper cells in infected WT and Bam32^-/-^ mice at all times after infection (Fig 5D) despite the significant differences in germinal centre B cells, suggesting that the effects of Bam32 deficiency may be intrinsically restricted to B cells in this model of infection.

Next, we assessed whether the impaired germinal centre response in Bam32^-/-^ mice was also associated with impaired memory B cell response. At different times after infection, we gated on CD19^+^GL7^-^Fas^-^ cells and assessed their co-expression of PD-L2 and CD80 molecules (S2 Fig), markers previously used to delineate memory B cells [47]. As shown in (Fig 5G and 5H), although the frequency of PD-L2^+^CD80^+^ (memory B) cells increased in the spleens of infected WT and Bam32^-/-^ mice as the infection progressed, there was no difference between the two mouse strains. Collectively, these results suggest that the defective parasite-specific IgG responses observed in *T*. *congolense*-infected Bam32^-/-^ mice may be related to the impaired germinal centre B cell response.

### Impaired parasite control in B cell deficient mice reconstituted with Bam32^-/-^ B cells

**Fig 6:**
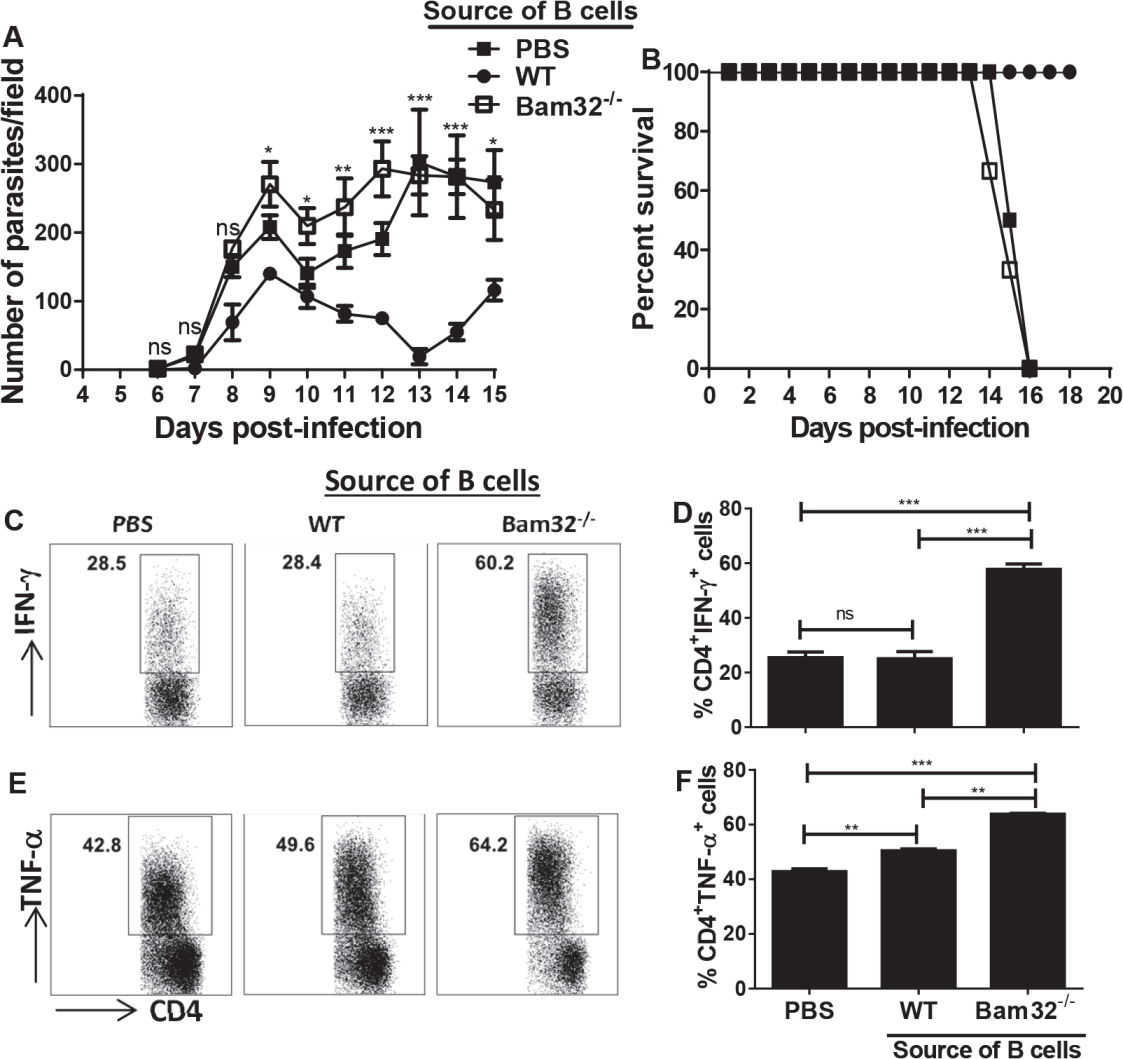
Bam32^-/-^ B cells do not mediate parasite control in B cell deficient mice. B cells were isolated from naïve WT and Bam32^-/-^ mice and adoptively transferred intravenously into μMT mice. Control mice received PBS. Forty-eight hours after transfer, recipient mice were infected with 10^3^ *T*. *congolense*, parasitemia (A) and survival (B) were monitored. At sacrifice, spleen cells were stimulated directly *ex-vivo* with PMA, BFA and ionomycin for 3–5 hr, stained and assessed for intracellular cytokine expression by flow cytometry. Shown are representative dot plots (C and E) and bar graphs (D and F) showing the means +/- standard error of the percentages of CD4^+^ T cells that express IFN-γ (C and D) and TNF-α (E and F). Data shown are representative of 2 separate experiments (n = 3 mice per experiment) with similar outcome. *, p <0.05; **, p < 0.01; ***, p < 0.001; ns, not significant.

Although the preceding observations strongly suggest that impaired B cells responses may be primarily responsible for the enhanced susceptibility of Bam32^-/-^ mice to infection, it is plausible that other cells may also be important. This is because the Bam32^-/-^ mice used in our studies are globally deficient in Bam32 signalling in other immune cells including T cells. Therefore, we wished to determine whether the enhanced susceptibility of Bam32^-/-^ mice is related to primary defects in their B cells. We transferred WT and Bam32^-/-^ B cells into μMT mice (which have intact T cells) and infected them with *T*. *congolense*. Results presented in (Fig 6) show that μMT mice that received Bam32^-/-^ B cells failed to control first wave of parasitemia (Fig 6A) and succumbed to the infection within 15 days (Fig 6B) akin to μMT mice given only PBS. In contrast, μMT mice that received WT B cells controlled their first wave of parasitemia and survived until the termination of the experiment (Fig 6A and 6B). Interestingly, CD4^+^ T cells from μMT mice that received Bam32^-/-^ B cells produced significantly (p < 0.01–0.001) more IFN-γ (Fig 6C and 6D) and TNF-α (Fig 6E and 6F) compared to the group that received WT B cells or PBS. Taken together, these results indicate that intrinsic B cell defect is primarily responsible for the enhanced susceptibility of Bam32^-/-^ mice to *T*. *congolense* infection.

## Discussion

The primary objective of this study was to investigate the role of Bam32, a B cell adaptor molecule critical for BCR signalling and antibody responses, in experimental African Trypanosomiasis in mice. We found that deficiency of Bam32 results in failure to control parasitemia during the chronic phase of the disease, leading to significantly decreased survival in an otherwise relatively resistant strain of mice. This was associated with increased production of disease-exacerbating proinflammatory cytokines (IFN-γ, IL-16 and TNF-α), impaired production of parasite-specific IgG, IgG1 and IgG2a antibodies and failure to sustain strong germinal centre responses during the chronic phase of infection. Since effective clearance of parasitemia is mediated by IgG antibodies against the variant surface glycoprotein and common antigens [28] and death of infected mice is usually associated with overproduction of proinflammatory cytokines, it is conceivable that impaired production of IgG antibodies and high production of proinflammatory cytokines contribute to the susceptibility of Bam32^-/-^ mice to *T*. *congolense* infection. To the best of our knowledge, this is the first report showing the contribution of Bam32 in resistance to a protozoan parasite.

The susceptibility to *T*. *congolense* infection in mice has been associated with several factors, including immunosuppression [48–51], systemic inflammatory response syndrome resulting from cytokine storm [14–17], impaired antibody response [28,52,53], induction of regulatory T cells [41,43], and hepatotoxicity particularly during the chronic phase of the disease [36]. We found that infected Bam32^-/-^ mice were unable to control chronic (late stage) parasitemia and show shorter survival time than their wild type counterpart mice and this was associated with significantly increased splenomegaly and hepatomegaly. However, serum levels of ALT and AST were not different, suggesting that hepatotoxicity may not account for the increased susceptibility of Bam32^-/-^ mice to *T*. *congolense* infection. Interestingly, cells from infected Bam32^-/-^ mice produced significantly higher amounts of disease exacerbating proinflammatory cytokines (including IFN-γ, TNF-α and IL-6), suggesting that the uncontrolled production of these cytokines may be related to death of infected Bam32^-/-^ mice.

Studies on the role of Bam32 in proinflammatory cytokine production are limited. A study by Sommers et al showed that Bam32 deficiency does not affect CD4^+^ T cell proliferation and their production of IL-17 and TNF-α. However and consistent with our results, there was a trend of increased IFN-γ following polyclonal stimulation with anti-CD3 and anti-CD28 mAbs [27]. We found that spleen cells from infected Bam32^-/-^ mice produced significantly higher amount of proinflammatory cytokines (IFN-γ, TNF-α and IL-6) following *in vitro* restimulation, suggesting that Bam32 might act as a negative regulator of these cytokines in the context of *T*. *congolense* infection. The differences in the effects of Bam32 deficiency on cytokine production might be related to differences in the experimental models. The studies of Sommers et al focused primarily on T cells from uninfected mice following polyclonal stimulation with anti-CD3 and anti-CD28 mAbs whereas our studies were carried out under parasitic infection condition using unfractionated spleen cells. Splenic and hepatic macrophages are producers of proinflammatory cytokines (including IL-6 and TNF-α) [13,54], and plastic-adherent T cells that exhibit macrophage-like properties are the major producers of IFN-γ in *T*. *congolense*-infected mice [16]. It is conceivable that signalling via Bam32 suppresses cytokine production in immune cells. In line with this, we found that the production of IFN-γ and TNF-α by CD4^+^ T cells from μMT mice that received Bam32^-/-^ B cells was significantly higher than those that received WT B cells or PBS (Fig 6C–6F). This suggests that signalling via Bam32 could endow B cells with the ability to downregulate cytokine production in CD4^+^ T cells.

Previous reports have linked Tregs with susceptibility to experimental *T*. *congolense* infection in mice, the mechanism possibly being through the production of IL-10 to dampen immune response [43,54]. We found no difference in the frequency of Tregs in spleens of infected WT and Bam32^-/-^ (Fig 3A and 3B) mice, suggesting that Bam32 does not influence the expansion and/or survival of Tregs in mice. However, spleens of infected Bam32^-/-^ mice contained significantly higher numbers of Tregs than those of WT counterpart mice, due primarily to increased splenomegaly. The increase in absolute numbers of Tregs in infected Bam32^-/-^ mice suggests that Tregs might contribute to the enhanced susceptibility of these mice to *T*. *congolense*.

B cells are critical for clearance of parasitemia and survival in murine experimental African trypanosomiasis. *T*. *congolense* infection has been shown to cause depletion of several B cell subsets [55] and B cell deficient mice are highly susceptible to various strains of African trypanosomes [56]. The susceptibility of B cell deficient mice to *T*. *congolense* infection is reversed by passive transfer of VSG-specific antibody or primed B cells [56]. In line with this, we found that μMT mice were unable to control their first wave of parasitemia and succumbed within 15 days post-infection. While adoptive transfer of B cells from WT mice resulted in effective parasite control and survival, the transfer of B cells from Bam32^-/-^ mice did not result in parasite control in μMT mice. In a previous study where we mixed and adoptively transferred equal numbers of WT and Bam32^-/-^ B cells into μMT mice, we showed that Bam32 KO B cells do not have an engraftment disadvantage, but rather have some proliferative advantage over WT B cells in the recipient μMT mice [44]. This suggests that the differences in parasitemia and survival observed here were not related to poor engraftment of Bam32^-/-^ B cells in μMT mice. Collectively, our results suggest that intrinsic B cell defects may be primarily responsible for the enhanced susceptibility of Bam32^-/-^ mice to *T*. *congolense* infection. VSG-specific antibodies mediate complement-mediated lysis *in vitro* [57,58], agglutination [59], immobilization [60] and increased uptake of trypanosomes by macrophages [61,62]. Although both IgM and IgG antibody subclasses have been shown to mediate anti-trypanosome clearance [63], it is generally accepted that the different IgG antibody classes are more important than IgM in mediating parasite control and survival of trypanosome-infected mice [28,64]. Thus, although serum levels of trypanosome-specific IgM antibodies were comparable in infected WT and Bam32^-/-^ mice, the impaired production of parasite-specific IgG, IgG1 and IgG2a antibody classes in infected Bam32^-/-^ mice could be responsible for their enhanced susceptibility to infection. Interestingly, IgG levels were most significantly decreased at later time points (from day 28 post-infection), suggesting a failure to sustain protective antibody responses over time.

The crosslinking of B cell receptors by their cognate antigens leads to the generation of intracellular signalling events that ultimately result in B cell activation. The activated B cells migrate to the lymphoid follicles where they undergo extensive proliferation and differentiation into antibody-producing plasma cells. These follicular areas of extensive B proliferation and differentiation (also called germinal centres) are critically important for somatic hypermutation, class-switching and affinity maturation events that are dependent on cognate interaction with follicular CD4^+^ T helper cells. We found that Bam32^-/-^ mice have impaired germinal centre B cell response starting from day 28 post-infection, a time that correlated with onset of uncontrolled parasitemia, hepatomegaly, splenomegaly and lower serum levels of IgG, IgG1 and IgG2a in infected Bam32^-/-^ mice. Interestingly, there was no significant difference in germinal centre B cells numbers between infected WT and Bam32^-/-^ mice early in the infection, suggesting that premature germinal centre crash occurs in infected Bam32^-/-^ mice. In support of this, both flow cytometry and immunofluorescence staining clearly revealed germinal centre deterioration by day 48 post-infection in infected Bam32^-/-^ mice. This is consistent with a previous study that showed germinal centre collapse in Bam32^-/-^ mice after immunization with ova/alum [44].

Surprisingly, despite the significant differences in germinal centre B cells, we found no differences in the percentages or absolute numbers of T follicular helper cells in the spleens of infected WT and Bam32^-/-^ mice throughout the course of infection. In fact, Bam32^-/-^ mice had higher T follicular cells at the later phase of infection, suggesting that the suboptimal GC response is not related to intrinsic defects in T follicular helper cells numbers and/or function. Collectively, these observations suggest that the impaired IgG response in infected Bam32^-/-^ mice may be due to B cell intrinsic defects as described previously [21,23,44]. When memory B cell subsets in these mice were assessed, we found as expected that the frequency of memory B cell population (i.e. CD19^+^GL7^-^Fas^-^CD80^+^PD-L2^+^) increased in the spleens of infected WT and Bam32^-/-^ mice as the infection progressed (see S2 Fig for gating strategy). However, there was no difference in the frequency of memory B cells in the spleens of infected WT and Bam32^-/-^ mice at different times after infection, suggesting that differences in generation of memory B cells could not account for the enhanced susceptibility of infected Bam32^-/-^ mice. Collectively, the new set of data support our conclusion that the susceptibility of Bam32^-/-^ mice to *T*. *congolense* infection is due in part to their inability to mount a strong and sustainable germinal centre response, which ultimately results in impaired parasite-specific antibody production.

In conclusion, we have demonstrated that Bam32 is an important molecule that contributes to optimum resistance to experimental *T*. *congolense* infection in mice. Deficiency of this adaptor molecule negatively impacts on parasite-specific germinal centre formation and IgG responses *in vivo*. In addition, it dramatically enhances proinflammatory cytokine production, suggesting that Bam32 may act as a negative regulator of proinflammatory cytokine gene expression. Collectively, these findings identify Bam32 as an indispensable molecule for optimal anti-trypanosome IgG antibody response and suppression of disease-promoting proinflammatory cytokines and its deficiency leads to inability to control *T*. *congolense* infection in mice.

## Supporting Information

S1 Fig CD3^+^ T cells are the major producers of cytokines after *T*. *congolense* infection. WT mice were infected with 10^3^ *T*. *congolense* and at indicated times sacrificed and their spleen cells were stimulated directly *ex-vivo* with PMA, BFA and ionomycin for 3–5 hr, stained for intracellular expression of IFN-γ TNF-α and IL-10 and assessed by flow cytometry. Representative dot plots (A, C and E) and bar graphs showing the means +/- standard error of the percentages (B, D and F) of CD3^+^ T cells that express IFN-γ (A and B), TNF-α (C and D) and IL-10 (E and F) are shown. Results are representative of 2 different experiments (n = 3–5 mice per experiment) with similar outcome. ns, not significant; *, p < 0.05; **, p < 0.01; ***, p < 0.001.
(TIF)

S2 Fig Gating strategy for memory B cells. To assess memory B cell subset, splenocytes were first gated on CD19^+^ (B cells) and then gated on GL7^-^ and FAS^-^ (double negative) population to exclude the germinal centre B cells. Memory B cells were then assessed on expression of CD80 and PD-L2 (double positive) B cells.
(TIF)
