# Supplementary figures and images for: The B Cell Adaptor Molecule Bam32 Is Critically Important for Optimal Antibody Response and Resistance to Trypanosoma congolense Infection in Mice
Source: PLoS Negl Trop Dis. 2015 Apr 13;9(4):e0003716. doi: 10.1371/journal.pntd.0003716 (PMC4395458; doi:10.1371/journal.pntd.0003716)

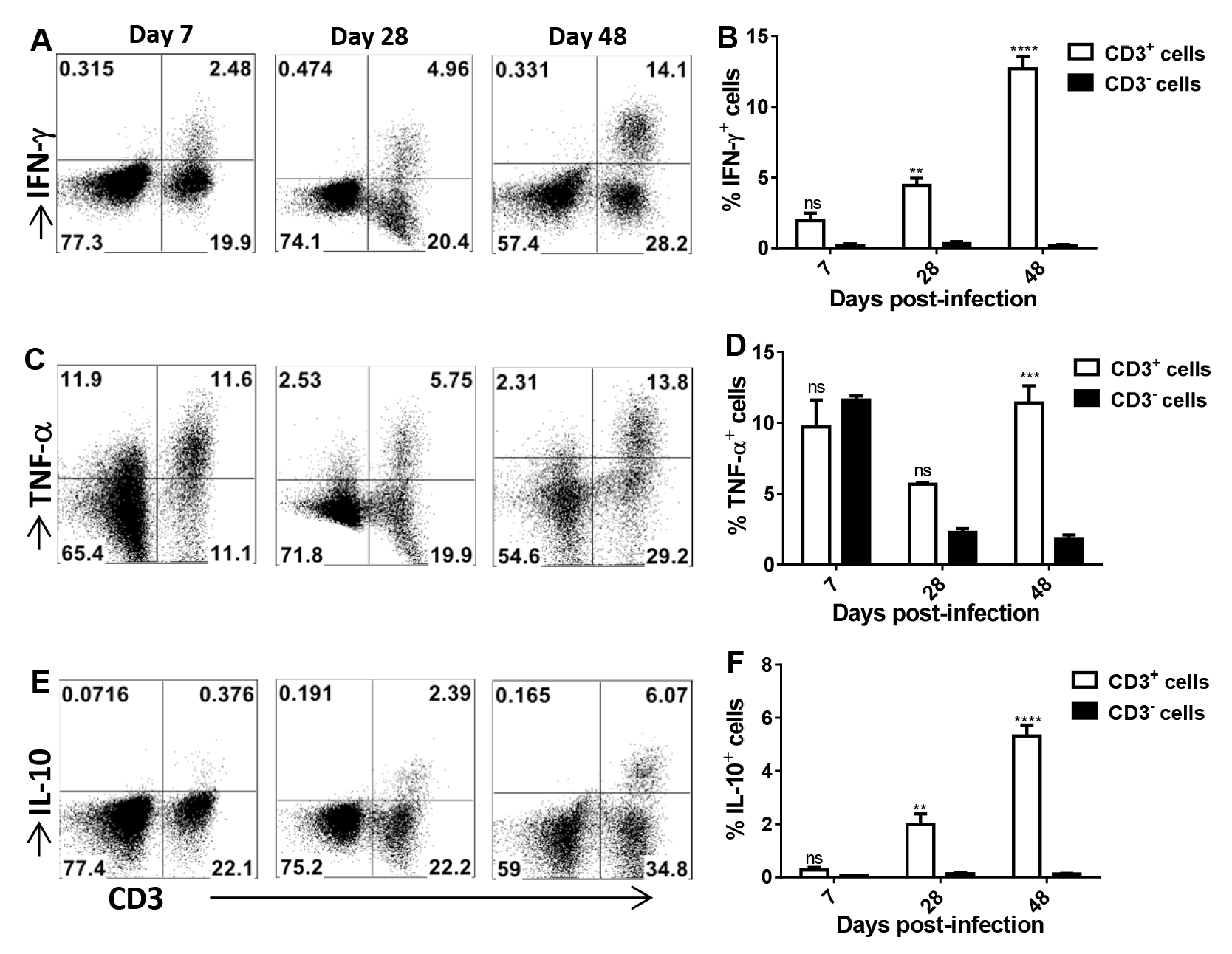

Supplement: S1 Fig — WT mice were infected with 103 T. congolense and at indicated times sacrificed and their spleen cells were stimulated directly ex-vivo with PMA, BFA and ionomycin for 3–5 hr, stained for intracellular expression of IFN-γ TNF-α and IL-10 and assessed by flow cytometry. Representative dot plots (A, C and E) and bar graphs showing the means +/- standard error of the percentages (B, D and F) of CD3+ T cells that express IFN-γ (A and B), TNF-α (C and D) and IL-10 (E and F) are shown. Results are representative of 2 different experiments (n = 3–5 mice per experiment) with similar outcome. ns, not significant; *, p < 0.05; **, p < 0.01; ***, p < 0.001. (TIF) [file pntd.0003716.s001.tif]

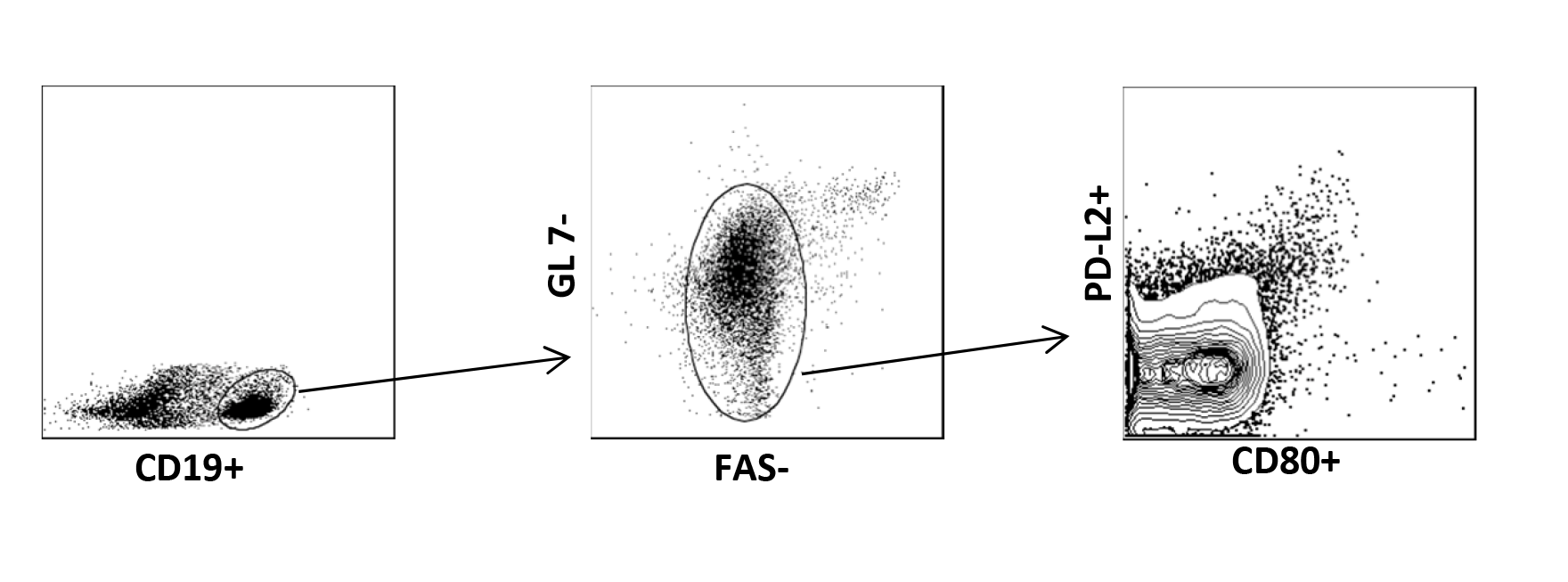

Supplement: S2 Fig — To assess memory B cell subset, splenocytes were first gated on CD19+ (B cells) and then gated on GL7- and FAS- (double negative) population to exclude the germinal centre B cells. Memory B cells were then assessed on expression of CD80 and PD-L2 (double positive) B cells. (TIF) [file pntd.0003716.s002.tif]
